# Knowledge, attitudes and practices regarding tuberculosis amongst healthcare workers in Moyen-Ogooué Province, Gabon

**DOI:** 10.1186/s12879-021-06225-1

**Published:** 2021-05-27

**Authors:** Anja Vigenschow, Jean Ronald Edoa, Bayode Romeo Adegbite, Pacome Achimi Agbo, Ayola A. Adegnika, Abraham Alabi, Marguerite Massinga-Loembe, Martin P. Grobusch

**Affiliations:** 1 Centre de Recherches Médicales de Lambaréné and African. Partner Institution, German Center for Infection Research, Lambaréné, Gabon; 2 Institute of Tropical Medicine, Tübingen University, Tübingen, Germany; 3 Center of Tropical Medicine and Travel Medicine, Department of Infectious Diseases, Amsterdam University Medical Centers, location AMC, Amsterdam Infection & Immunity, Amsterdam Public Health, University of Amsterdam, Amsterdam, The Netherlands

**Keywords:** Tuberculosis, Infection control, Healthcare workers, Knowledge, Attitudes, Practices, Gabon

## Abstract

**Background:**

In countries with a high tuberculosis incidence such as Gabon, healthcare workers are at enhanced risk to become infected with tuberculosis due to their occupational exposure. In addition, transmission can occur between patients and visitors, if a tuberculosis infection is not suspected in time. Knowledge about tuberculosis and correct infection control measures are therefore highly relevant in healthcare settings.

**Methods:**

We conducted an interviewer-administered knowledge, attitude and practice survey amongst healthcare workers in 20 healthcare facilities at all levels in the Moyen-Ogooué province, Gabon. Correctly answered knowledge questions were scored and then categorised into four knowledge levels. Additionally, factors associated with high knowledge levels were identified. Fisher’s Exact test was used to identify factors associated with high knowledge levels.

**Results:**

A total of 103 questionnaires were completed by various healthcare personnel. The most-frequently scored category was ‘intermediate knowledge’, which was scored by 40.8% (42/103), followed by ‘good knowledge’ with 28.2% (29/103) and ‘poor knowledge’ with 21.4% (22/103) of participating healthcare workers, respectively. ‘Excellent knowledge’ was achieved by 9.7% (10/103) of the interviewees. Apart from the profession, education level, type of employing healthcare facility, as well as former training on tuberculosis were significantly associated with high knowledge scores.

Attitudes were generally positive towards tuberculosis infection control efforts. Of note, healthcare workers reported that infection control measures were not consistently practiced; 72.8% (75/103) of the participants were scared of becoming infected with tuberculosis, and 98.1% saw a need for improvement of local tuberculosis control.

**Conclusions:**

The survey results lead to the assumption that healthcare workers in the Moyen-Ogooué province are at high risk to become infected with tuberculosis. There is an urgent need for improvement of tuberculosis infection control training for local healthcare personnel, particularly for less trained staff such as assistant nurses. Furthermore, the lack of adequate infection control measures reported by staff could possibly be correlated with a lack of adequate facility structures and protective equipment and requires further investigation.

**Supplementary Information:**

The online version contains supplementary material available at 10.1186/s12879-021-06225-1.

## Background

Healthcare workers (HCWs) play a fundamental role in the global fight against tuberculosis (TB). However, HCWs have a high risk of becoming infected with TB themselves, as they are often exposed to TB patients [1–3]. A recent meta-analysis found an approximately three-times higher incidence of active TB amongst HCWs as compared to the general population [4]. Occupational TB prevention and infection control (TBIC) measures in healthcare facilities are therefore of paramount importance.

In low-and-middle-income country (LMIC) settings such as Gabon, TBIC often poses challenges. The World Health Organization (WHO) recommends managerial, administrative and environmental controls that are feasible in all settings [5]. Those recommendations comprise, amongst others, the implementation of an infection control plan, triaging of presumed TB cases, regular TB screening amongst HCWs and optimising natural ventilation in the facility. In addition, the availability and correct use of particular respirators are an important personal protective measure.

In Gabon, TB is a significant public health problem and ranks amongst the main causes of death. In 2019, the prevalence of HIV was estimated at 2.35% (2348 cases per 100.000 population) [6]. The estimated incidence of TB was 521 cases per 100.000 population, with 22/100.000 population cases of multi-drug resistant (MDR-) and rifampicin-resistant (RR-) TB. The mortality rate was 147/100.000 population and therefore the third-highest in the world [7]. Thus, Gabon ranks amongst the countries with the highest TB incidence in the world. Regarding the high TB incidence and the absence of national TBIC guidelines at the time of the study, we assume that HCWs in Gabon are at high risk to become infected with TB, and thus to develop active disease. The situation is aggravated by a considerable HIV prevalence as well as a high proportion of MDR-TB [8–10]. At the time of the study, there was no systematic TB training program implemented for HCWs in Gabon.

For effective infection control and TB management, essential knowledge about TB is crucial. We therefore conducted a knowledge, attitude and practice (KAP) survey amongst HCWs in 20 healthcare facilities in Moyen-Ogooué province, Gabon, in order to assess HCWs awareness of the disease and to gain insight into currently practiced infection control measures.

## Methods

### Study population

Participants were included at 20 individual healthcare facilities at different levels of the healthcare pyramid in Moyen-Ogooué province. These included the two hospitals of Lambaréné (one public and one private), the province’s capital, an ambulatory HIV clinic and 17 rural dispensaries. Despite being chosen conveniently, the sites provide a fair representation of all primary and intermediate healthcare services of Moyen-Ogooué province. In the two hospitals, participants were recruited at departments that were considered as high-risk areas of TB transmission, including the outpatient, radiology and emergency departments as well as the internal medicine and paediatric wards. Each department as well as the HIV clinic was visited on two to three days, and all employees present during this period were asked to participate. Due to their small number of staff, dispensaries were visited on a single day only.

### Survey questionnaire

We conducted the KAP survey based on an interviewer-administered questionnaire, which was specifically designed for the study. Questions were partly based on a TB KAP survey manual [11]. The questionnaire was validated in a pilot study with 10 HCWs from the local research center, and adjusted accordingly. The survey comprised 11 socio-demographic variables, 16 knowledge questions including six questions with multiple possible correct answers, nine attitude questions and 10 practice questions. The questionnaire is provided as Supplement 1.

### Study procedures

Interviews were held between November 2016 and March 2017. Open questions were read out to the interviewees, and, if necessary, non-leading explanations were given. All interviews were held by the same investigator during working hours in the respective facility. Answers given to the open questions were then documented in terms of pre-defined categories. Each correct answer given to a knowledge question was scored 1. The maximum score achievable was 27. Knowledge levels were categorised as ‘excellent’ (> 80% correct answers), ‘good’ (> 60–80%), ‘intermediate’ (> 40–60%) and ‘poor’ (< 40%). Attitudes and practices were collected as qualitative data only.

### Statistical analysis

Knowledge levels amongst different healthcare workers were compared using cross-tabulation. Given the small numbers, Fisher’s exact test, was used to assess the correlation of influencing factors with knowledge levels. Statistical analysis was performed with IBM SPSS (Version 24). The *p*-value of 5% was considered statistically significant.

### Ethical considerations

All methods were performed in accordance with the relevant guidelines and regulations. Survey participation was voluntary, and written informed consent was obtained from all participants prior to the interviews. The questionnaires were completely anonymous and did not contain any personal identifying information. Authorisation was obtained from the regional health director of Moyen-Ogooué province as well as the institutional ethics committee of CERMEL (reference number: CEI-CERMEL 008/2016).

## Results

### Study population characteristics

Out of the 103 questionnaires, 68 (66.0%) were completed at the two hospitals, followed by 25 (24.3%) at the dispensaries and 10 (0.7%) at the regional HIV clinic. Interviewees represented a cross-section of the various relevant health professions involved (Table 1). Work experience of the interviewees across professions ranged from one month to 38 years (mean 11.5 years; SD = 7.6).

**Table 1:** Knowledge levels by profession (*n* = 103)

			Knowledge Levels				Total
			Poor	Intermediate	Good	Excellent	
**Profession**	Auxiliary nurse	*N*	5	2	1	0	8
		%	62.5	25.0	12.5	0.0	100.0
	Assistant nurse	N	11	30	16	1	58
		%	19.0	51.7	27.6	1.7	100.0
	Nurse	N	1	3	5	2	11
		%	9.1	27.3	45.5	18.2	100.0
	Doctor	N	0	0	3	7	10
		%	0.0	0.0	30.0	70.0	100.0
	Other	N	5	7	4	0	16
		%	31.2	43.8	25.0	0.0	100.0
**Total**		N	22	42	29	10	103
		%	21.4	40.8	28.2	9.7	100.0

### Knowledge levels

Table 1 displays knowledge levels of different groups of HCWs. The category ‘intermediate knowledge’ comprises 42 HCWs and therewith the largest number of respondents (40.8%). This was followed by ‘good knowledge’ with 29 (28.2%) HCWs. The knowledge category ‘poor’ was scored by 22 (21.4%) respondents. Ten HCWs (9.7%) achieved ‘excellent’ knowledge scores in the questionnaire.

The mean knowledge score was 10 (SD = 3.7) for auxiliary nurses, 14.2 (SD = 1) for assistant nurses, 17.7 (SD = 2.8) for nurses, 22.4 (SD = 1.4) for physicians, and 12.5 (SD = 2.7) for other HCWs.

### Factors associated with knowledge levels

Since not all subgroups included physicians, who had overall good or excellent knowledge, Fisher’s exact test was only conducted on the other 93 HCWs.

As presented in Table 2; age, gender, work experience, and family TB cases did not have any significant influence on knowledge about TB. Statistically significant associations with higher knowledge levels were found regarding the level of education (*p* = 0.001), type of healthcare facility (*p* = 0.018), and former TB training (*p* = 0.001).

**Table 2:** Factors associated with knowledge levels of HCWs other than physicians (*n* = 93)

	Knowledge Levels			*p*-value
	Poor (*n* = 22)	Intermediate (*n* = 42)	Good/Excellent* (*n* = 29)	
Age				0.096
≤ 30 years	3 (13.6%)	9 (21.4%)	1 (3.4%)	
31–40 years	7 (31.8%)	12 (28.6%)	16 (55.2%)	
> 40 years	12 (54.5%)	21 (50.0%)	12 (41.4%)	
Gender				0.351
F	17 (77.3%)	25 (59.5%)	18 (62.1%)	
M	5 (22.7)	17 (40.5%)	11 (37.9%)	
Level of education				**0.001**
CEP	5 (22.7%)	9 (21.4%)	0 (0.0%)	
BEPC	11 (50.0%)	28 (66.7%)	17 (58.6%)	
BAC	2 (9.1%)	3 (7.1%)	8 (27.6%)	
University	0 (0.0%)	2 (4.8%)	2 (6.9%)	
Other	4 (18.2%)	0 (0.0%)	2 (6.9%)	
Facility				**0.018**
Hospital	12 (54.5%)	30 (71.4%)	26 (89.7%)	
Dispensary	10 (45.5%)	12 (28.6%)	3 (10.3%)	
Work experience				0.245
≤ 5 years	7 (31.8%)	12 (28.6%)	3 (10.3%)	
6–10 years	8 (36.4%)	8 (19.0%)	11 (37.9%)	
11–20 years	6 (27.3%)	17 (40.5%)	12 (41.4%)	
> 20 years	1 (4.5%)	5 (11.9%)	3 (10.3%)	
Family TB				0.544
No	16 (72.7%)	25 (59.5%)	20 (69.0%)	
Yes	6 (27.3%)	17 (40.5%)	9 (31.0%)	
TB training				**0.001**
No	21 (95.5%)	25 (59.5%)	15 (51.7%)	
Yes	1 (4.5%)	17 (40.5%)	14 (48.3%)	

*Since the category “excellent” contains only three individuals, the two categories were pooled together for the analysis

*CEP* Certificat d’ètudes primaires (primary school certificate), *BEPC* Brevet d’Études du Premier Cycle (General Certificate of Secondary Education), *BAC* baccalauréat (general qualification for university entrance)

### Attitudes

Answers given by HCWs to questions about their attitude towards TB are presented in Table 3. While 84 HCWs (81.6%) affirmed that they could get TB themselves, 15 HCWs (14.6%) claimed that they could not be infected. The explanations provided for this assumption were: (1) BCG vaccination (*n* = 7; 46.7%); (2) lack of exposure due to sufficient precautions (*n* = 4; 26.7%).

**Table 3:** Attitudes of HCWs towards TB (*n* = 103)

	Yes	No	Not sure
	*N* (%)	*N* (%)	*N* (%)
Do you think you could get TB?	84 (81.6)	15 (14.6)	4 (3.9)
Are you scared of getting TB?	75 (72.8)	25 (24.3)	3 (2.9)
Would you continue to socialize with your friend, if he was diagnosed with TB?	97 (94.2)	3 (2.9)	3 (2.9)
Would you share the same cutlery, plates and glasses with a family member, if he was infected with TB?	11 (10.7)	91 (88.3)	1 (1.0)
Would you say that TB is a stigmatized disease?	65 (63.1)	33 (32.0)	5 (4.9)
Would you like to learn more about TB?	101 (98.1)	1 (1.0)	1 (1.0)
Would you be willing to get tested for TB regularly?	100 (97.1)	2 (1.9)	1 (1.0)
Do you feel TB is major threat to public health in Gabon?	98 (95.1)	3 (2.9)	2 (1.9)
Do you think there is a need of improvement in TB control in your region?	101 (98.1)	1 (1.0)	1 (1.0)

The majority (*n* = 75, 72.8%) was scared of getting TB and named the following reasons (multiple answers were possible): (1) severity of active TB infection (*n* = 35, 46.7%); (2) risk of infecting a family member (*n* = 21, 28.0%); and (3) long duration and/or adverse effects of TB treatment (*n* = 20; 26.7%).

Fear of death, social isolation and irreversible damage to the lung were further reasons given by the respondents.

Almost all HCWs (*n* = 97; 94.2%) said that they would keep seeing a friend who was diagnosed with TB; however, 91 (88.3%) would not continue to use the same cutlery, plates and glasses if someone in their family was infected with TB. TB was considered as a stigmatising disease by 65 HCWs (63.1%).

Except for two HCWs, all respondents said that they wished to learn more about TB; and 100 (97.1%) would approve to be screened regularly for TB. Most HCWs (*n* = 98; 95.1%) considered TB as a major public health problem in Gabon; and 101 (98.1%) saw a need for improvement in regional TB control. The following three measures were the most-frequently suggested ideas to improve general TB control (multiple answers possible): (1) conduction of awareness-raising campaigns (*n* = 70; 68,0%); (2) intensifying case finding (*n* = 16; 15.5%); and (3) vaccination programs (*n* = 10; 9.7%).

#### Practices

Table 4 presents various TB infection control measures and the number of HCWs who reported to practice them. The majority (*n* = 82; 79.6%) would suspect TB infection in a patient presenting with a chronic cough, and 50 (48.5%) would separate them from other patients. About half of the interviewed HCWs (*n* = 56; 54.4%) reported to wear a face mask when being with a coughing patient, whereas 73 (70.9%) would wear one when TB infection was confirmed. Ensuring cough hygiene by providing a mask to coughing patients was named by 55 (53.4%).

**Table 4:** TB infection control practices reported by HCWs (n = 103)

	Practiced *N* (%)
Suspecting TB in patients with chronic cough	82 (79.6)
Separating coughing patients from others	50 (48.5)
Wearing a mask when with a coughing patient	56 (54.4)
Wearing a mask when with a TB patient	73 (70.9)
Providing a mask to a coughing patient	55 (53.4)
Increase natural ventilation	69 (67.0)
Turning on a fan	16 (15.5)
Prioritization of coughing patients	32 (31.1)
Educating patients about TB	63 (61.2)
Educating patients about cough hygiene	77 (74.8)

In order to increase the natural ventilation as an environmental TB control measure, 69 (67%) of the respondents would open windows and doors and only 16 (15.5%) would make use of a fan. Triage by prioritising coughing patients was reported to be practiced by 32 (31.1%) HCWs.

Patient education in terms of providing information about TB to newly diagnosed TB patients was practiced by 63 (61.2%) of the HCWs, and 77 (74.8%) would give cough hygiene instructions to patients with a cough.

## Discussion

### Knowledge levels

This study provides insight into the knowledge levels about TB of all kinds of HCWs in the Moyen-Ogooué province and can help to elaborate future training programs. In their study from South Africa, Naidoo et al. showed that training programs for HCWs can increase their knowledge levels significantly [12].

Not surprisingly, well-trained HCWs with a university degree or state’s diploma had good and excellent knowledge about TB. However, they represent the smallest portion of personnel working in regional healthcare facilities. Almost two-thirds of the respondents were auxiliary or assistant nurses, who had significantly lower knowledge levels. In conclusion, as knowledge about TB is evolving, TB training updates should be made available to all levels of HCWs; however, the emphasis of the training efforts should be on focused need-tailored TB training for HCWs with lower professional degrees and thus facilitate improvements for the broad majority of their staff.

### Factors associated with good knowledge

The study identified factors associated with good knowledge levels in order to determine which group of HCWs needs to be focused on. The professional level, and accordingly, the highest level of education, were both significantly associated with good knowledge about TB. In addition, HCWs who had received former TB training, irrespective of the extent of training, had significantly higher knowledge levels than their colleagues. Work experience, however, was not statistically associated with good knowledge about TB. These findings empathise the importance of regular training.

HCWs in hospitals had generally better knowledge levels than in dispensaries, which can lead to the assumption that primary healthcare services, particularly in rural areas, are often neglected in training programs.

These findings are comparable to the factors identified in a KAP survey conducted amongst healthcare workers in Mozambique, which also showed an association of knowledge scores with the level of education and profession, while gender and age did not have any significant influence [13].

### Attitudes

The fact that the majority of the participants would not share the same cutlery, plates and glasses with a TB infected family member implies that there are common knowledge gaps about the ways of TB transmission. This can possibly promote stigmatisation and social exclusion of TB-infected individuals. The idea that TB can be transmitted by eating from the same plate was also found in other surveys [14, 15].

Almost two-thirds of the HCWs considered TB as a stigmatising disease. A study with TB patients in Lambaréné from 2013 found similar perceptions, as the majority of the patients had experienced stigma themselves [16]. Nevertheless, almost all HCWs would agree to get themselves tested for TB regularly. In addition, most respondents stated that they would like to learn more about TB. Studies from other countries have shown a similar positive attitude of HCWs towards TBIC [17].

Most respondents saw a need for improvement of regional TB control and perceived it as a major public health threat. The high awareness amongst HCWs of TB being a serious health issue in their region is an optimal pre-condition for new TBIC policies implementation as well as training programs. The fact that 72.8% of HCWs were scared of becoming infected with TB emphasises even more the urgent need for action.

### Good practice

The reasons why certain TBIC measures were practiced or not were not implied in the survey. The absence of resources such as appropriate respirators or fans was not taken into account in the questionnaire.

About half of the respondents said that they would wear a mask when dealing with a coughing patient. However, since appropriate respirators were unavailable in most facilities, HCWs were referring to surgical masks, which are most appropriate when worn by the patient. A study from Uganda has shown that only a third of the HCWs knew that a surgical mask would not adequately protect them from getting TB from a patient [18]. Therefore, training programs should not only explain in what situation the use of a mask is adequate, but also which kind of mask would be the most appropriate for the patient and the attending HCW, respectively. Almost the same number of HCWs reported to provide coughing patients with a surgical mask. Due to the general lack of resources, particularly in primary healthcare facilities such as dispensaries, this is not surprising. On the other hand, it could also be attributed to the fear of stigmatising patients. As mentioned above, this could not be concluded from the survey and requires further investigation.

Nosocomial transmission is less likely if the time a TB patient spends in the facility is reduced to a minimum, which is one of the key recommendations of the so-called FAST strategy, an administrative approach to TBIC that is also feasible in LMIC settings [19]. The survey findings indicate that most HCWs were not aware of the importance of this measure, as less than a third of them reported to prioritise coughing patients in the admission process. Once implemented into the patient management routine, this TBIC measure can prevent transmission within the facility, particularly in waiting areas.

Only a minority reported to turn on a fan in order to direct the airflow away from them when receiving a TB patient, as it is recommended by WHO [20]. However, fans were unavailable in most facilities. Despite the economical advantage and high effectiveness of natural ventilation compared to mechanical ventilation [21], only about two-thirds of the interviewees reported to open doors and windows when dealing with an infectious TB patient. This possibly indicates a general lack of awareness towards environmental measures.

### Strengths and limitations

Several KAP surveys relating to TB control have been conducted all over the world. However, this was the first survey conducted amongst HCWs in Gabon. Although the sample size is relatively small, the study population represents the full range of healthcare workers in the Moyen-Ogooué province. Another strength of the study was that the survey was carried out as personal interviews and thus had a maximum response rate. The interviews were all carried out by the same interviewer, ensuring that questions were asked in the same way, leading to comparable responses. Most surveys assessing knowledge levels about TB were designed as self-administered multiple-choice questions, allowing to guess the correct answer. Due to the open question format, the survey allowed insight into the actual awareness level of HCWs about TB.

### General conclusions

There is no data available regarding the actual TB incidence amongst HCWs in Gabon. However, the survey findings suggest that due to a lack of general knowledge about TBIC, they are not adequately protected. However, HCWs seemed to be generally motivated for training and open for screening procedures amongst themselves, even though many perceived TB as a stigmatising disease.

### Way forward

Standardized TBIC training of HCWs should be implemented on a national level with focus on assistant nurses, as they represent the primary healthcare level in Gabon. Another crucial step is the implementation of administrative measures in the daily routine work. In order to improve practices, resources for effective TBIC need to be available in all facilities, particularly personal protective equipment such as N-95 respirators for HCWs and a sufficient supply of surgical masks for TB patients. Although stigma did not seem to be problematic amongst HCWs themselves, awareness campaigns should also be conducted amongst the general population, as misbeliefs about TB increase stigma, thus hampering TB control efforts in the country.

## Supplementary Information

**Additional file 1: Supplement 1**: Study questionnaire.

## Data Availability

The datasets used and analysed in the framework of this study can be requested from the corresponding author.

## Abbreviations

CERMEL: Centre de Recherches Médicales de Lambaréné
HCWs: Healthcare workers
HIV: Human immunodeficiency virus
KAP: Knowledge, attitudes and practices
LMIC: Low-and-middle-income country
MDR-TB: Multi-drug resistant tuberculosis
TB: Tuberculosis
TBIC: Tuberculosis infection control
WHO: World Health Organization
