# Supplementary material for: Knowledge, attitudes and practices regarding tuberculosis amongst healthcare workers in Moyen-Ogooué Province, Gabon
Source: BMC Infect Dis. 2021 May 27;21:486. doi: 10.1186/s12879-021-06225-1 (PMC8157668; doi:10.1186/s12879-021-06225-1)
Supplement: Supplementary file 1 — Additional file 1: Supplement 1: Study questionnaire. [file 12879_2021_6225_MOESM1_ESM.docx]

**Supplement 1:**

Study questionnaire

**Knowledge, attitudes and practices regarding tuberculosis amongst healthcare workers in Moyen-Ogooué Province, Gabon**

*Authors*

Anja Vigenschow^1,2^, Jean Ronald Edoa^1^, Bayode Romeo Adegbite^1,3^, Pacome Achimi Agbo^1^, Ayola A. Adegnika^1,2^, Abraham Alabi^1^, Marguerite Massinga-Loembe^1,2^, Martin P. Grobusch^1,2,3*^

*Affiliations*

^1^Centre de Recherches Médicales de Lambaréné and African Partner Institution, German Center for Infection Research, Lambaréné, Gabon

^2^Institute of Tropical Medicine, Tübingen University, Tübingen, Germany

^3^Center of Tropical Medicine and Travel Medicine, Department of Infectious Diseases, Amsterdam University Medical Centers, location AMC, Amsterdam Infection & Immunity, Amsterdam Public Health, University of Amsterdam, Amsterdam, The Netherlands

*Correspondence: Prof. Martin P. Grobusch, Amsterdam University Medical Centers, location AMC, Meibergdreef 9,

**Demographics**

**Age**:

- ≤ 20 years
- 21-30 years
- 31-40 years
- 41-50 years
- > 50 years

**Gender:** 🞏 M 🞏 F

**Role in the facility (professional category of the current position):**

🞏 Doctor

🞏 Nurse

🞏 Assistant nurse

🞏 Midwife

🞏 Other:_______________

🞎 in training

**Level of education**:

🞏 CEP

🞏 BEPC

🞏 BAC

🞏 University degree (e.g. Master, Doctorate)

🞏 Other: _____________________

**Duration of work in health care:** ________ years

**Current department (if applicable):** _________________________

**Have you ever had TB?**

🞏 Yes 🞏 No

**Have you ever been tested for TB?**

🞏 Yes 🞏 No

**Has one of your family members had TB?**

🞏 Yes 🞏 No

**Are you vaccinated against TB (BCG vaccine)?**

🞏 Yes 🞏 No 🞏 Not sure

**Have you ever received training on TB during your work in the hospital?**

🞏 Yes 🞏 No 🞏 Not sure

If yes, what kind of training? ________________________________________________

**Knowledge:**

**1. What causes TB?**

- A bacterium
- I don’t know
- Other

**2. What are the typical symptoms that are used as an indicator for active pulmonary TB?**

- Cough
- Bloody sputum
- Fever
- Weight loss
- Night sweats
- I don’t know
- Other

**3. Can TB be completely cured?**

🞏 Yes 🞏 No 🞏 Not sure

**4. How is TB transmitted?**

- Through coughed-up droplets via the air
- I don’t know
- Other

**5. What is the standard treatment for TB?**

- Specific antibiotics/ anti-TB regimen/RHZE
- I don’t know
- Other

**6. How long does the standard treatment for drug-sensitive (TB responding well to therapy) TB of the lungs (pulmonary TB) usually take?**

- 6 months
- I don’t know
- Other

**7. What can happen if you take your TB drugs incorrectly/disrupt TB treatment? (more than one answer possible)**

- Development of resistances
- Relapse
- I don’t know
- Other

**8. Who has a higher risk to get TB than average population? (Name three risk groups/ risk factors)**

- People living with HIV/AIDS
- Young children
- Close contacts of a confirmed case
- Health care workers
- Laboratory staff
- People with medical conditions that weaken the immune system (e.g. Cancer, Diabetes)
- People under immunosuppressive therapy
- Undernourished people
- People drinking alcohol
- People smoking
- People using drugs
- Prisoners
- I don’t know
- Other

**9. Can you get TB when you are vaccinated with BCG?**

🞏 Yes 🞏 No 🞏 Not sure

**10. Is there a relationship between HIV and TB?**

🞏 Yes (go to 10.1) 🞏 No 🞏 Not sure

| **10.1. What is it?**   - A person with HIV is more likely to develop TB - I don’t know - Other |  |
| --- | --- |

**11. What is the most frequently used diagnostic laboratory test to diagnose TB in Gabon?**

- Sputum smear microscopy
- I don’t know
- Other

**12. What is the difference between latent TB infection and TB disease? (More than one answer possible)**

- TB infection is asymptomatic
- TB infection is not contagious
- I don’t know
- Other

**13. When you are in the same room with a TB-infected patient, how can you minimize the risk of getting TB from that patient? (more than one answer possible)**

- Increase airflow (e.g. open windows and doors, fan)
- Let patient perform cough hygiene (mask, tissue, arm, etc.)
- Wear respirator
- I don’t know
- Other

**14. What is MDR-TB?**

- TB that is resistant to the most important anti-TB drugs/ TB that doesn’t respond to the usual treatment
- I don’t know
- Other

**15. How can you get MDR-TB? (more than one answer possible)**

- Through infection from another person with MDR-TB
- Through incorrect treatment
- I don’t know
- Other

**16. Is MDR-TB curable?**

🞏 Yes 🞏 No 🞏 Not sure

**Attitude**

**1. Do you think you could get TB?**

🞏 Yes 🞏 No 🞏 Not sure

**If not, why?**_____________________________________________________

_______________________________________________________________

**2. Are you scared of getting TB?**

🞏 Yes 🞏 No 🞏 Not sure

**If yes, why?**_____________________________________________________

_______________________________________________________________

**3. Would you continue to socialize with your friend, if he was diagnosed with TB?**

🞏 Yes 🞏 No 🞏 Not sure

**4. Would you share the same cutlery, plates and glasses with a family member, if he was infected with TB?**

🞏 Yes 🞏 No 🞏 Not sure

**5. Would you say that TB is a stigmatized disease?**

🞏 Yes 🞏 No 🞏 Not sure

**6. Would you like to learn more about TB?**
🞏 Yes 🞏 No 🞏 Not sure

**7. Would you be willing to get tested for TB regularly?**

🞏 Yes 🞏 No 🞏 Not sure

**If not, why?**_____________________________________________________

_______________________________________________________________

**8. Do you feel TB is a major threat to public health in Gabon?**

🞏 Yes 🞏 No 🞏 Not sure

**9. Do you think there is a need of improvement in TB control in your region?**

🞏 Yes 🞏 No 🞏 Not sure

**If** **yes, how?**_____________________________________________________

_______________________________________________________________

**Practice**

**1. When a patient says he has been coughing for a month, I suspect TB**

🞎 Never 🞎Sometimes 🞎 Most of the times 🞎 Always

**2. I separate coughing patients from other patients**

🞎 Never 🞎Sometimes 🞎 Most of the times 🞎 Always

**3. When I deal with a coughing patient, I wear a mask**

🞎 Never 🞎Sometimes 🞎 Most of the times 🞎 Always

**4. When I deal with a confirmed active TB patient, I wear a mask**

🞎 Never 🞎Sometimes 🞎 Most of the times 🞎 Always

**5. I give patients who arrive with a cough a mask**

🞎 Never 🞎Sometimes 🞎 Most of the times 🞎 Always

**6. When a TB patient is in the room, I open windows and doors to increase natural ventilation**

🞎 Never 🞎Sometimes 🞎 Most of the times 🞎 Always

**7. When dealing with a TB patient, I turn on the fan to increase ventilation**

🞎 Never 🞎Sometimes 🞎 Most of the times 🞎 Always

**8. When a patient arrives at my facility with a cough, I prioritise him/her in comparison to patients without cough**

🞎 Never 🞎Sometimes 🞎 Most of the times 🞎 Always

**9. If a patient is newly diagnosed with TB, I give him/her information about his/her disease**

🞎 Never 🞎Sometimes 🞎 Most of the times 🞎 Always

**10. I explain coughing patients how they can practice cough hygiene, in order to avoid infecting other people around them**

🞎 Never 🞎Sometimes 🞎 Most of the times 🞎 Always
